# Prediabetes among HIV-infected individuals receiving antiretroviral therapy: prevalence, diagnostic tests, and associated factors

**DOI:** 10.1186/s12981-020-00284-1

**Published:** 2020-05-24

**Authors:** Angsana Phuphuakrat, Hataikarn Nimitphong, Sirimon Reutrakul, Somnuek Sungkanuparph

**Affiliations:** 1 Department of Medicine, Faculty of Medicine Ramathibodi Hospital, Mahidol University, Bangkok, Thailand; 2 Division of Endocrinology, Diabetes and Metabolism, University of Illinois at Chicago, Chicago, IL USA; 3 Chakri Naruebodindra Medical Institute, Faculty of Medicine Ramathibodi Hospital, Mahidol University, 111 Moo 14, Bang Pla, Bang Phli, Samut Prakan 10540 Thailand

**Keywords:** Antiretroviral therapy, Associated factors, Diagnostic test, HIV, Prediabetes, Prevalence

## Abstract

**Background:**

Metabolic complications in human immunodeficiency virus (HIV)-infected individuals are common. Prediabetes represents a high risk for future diabetes development. This study aimed to determine the prevalence, diagnostic methods, and associated factors of prediabetes among HIV-infected individuals receiving antiretroviral therapy (ART).

**Methods:**

A cross-sectional study was conducted among HIV-infected adults without a history of diabetes who were receiving ART. Fasting plasma glucose (FPG), 2-hour plasma glucose (2-h PG) after a 75-g oral glucose tolerance test, and hemoglobin A1c (HbA1c) were assessed.

**Results:**

A total of 397 patients with a mean age of 47.0 ± 9.8 years and 55.7% male, were studied. All received ART with undetectable plasma viral load. The mean duration of ART was 9.6 ± 5.2 years, and the mean CD4 cell count was 554 ± 235 cells/mm^3^. Among the patients, 28 (7.1%) had first-diagnosed diabetes, and 133 (33.5%) patients had prediabetes. Glycemia estimation by FPG, 2-h PG, and HbA1c showed a prediabetes prevalence of 17.4%, 14.7%, and 12.5%, respectively. The kappa statistics for the agreement of FPG and 2-h PG, HbA1c and 2-h PG, and HbA1c and FPG were 0.317, 0.429, and 0.396, respectively. In multivariate analysis, hypertension [odds ratio (OR) 3.38; 95% confidence interval (CI), 1.16-9.91; *p *= 0.026), and triglycerides > 150 mg/dL (OR 2.11; 95% CI, 1.01-4.44; *p *= 0.047) were factors significantly associated with prediabetes.

**Conclusions:**

Prediabetes among HIV-infected individuals receiving ART is common. The agreements of glycemia estimation methods are minimal to weak. HbA1c may underestimate prediabetes prevalence. Using FPG together with HbA1c increases the detection rate to approximately three-quarters of prediabetes patients. HIV-infected individuals who had hypertension and hypertriglyceridemia should be regularly assessed for prediabetes.

*Trial registration* ClinicalTrial.gov, NCT03545217. Registered 1 June 2018—Retrospectively registered, https://clinicaltrials.gov/ct2/show/NCT03545217

## Background

HIV infection is a major health problem worldwide, especially in low-to middle-income countries. The use of combination antiretroviral therapy (ART) has modified the natural history of HIV infection, leading to a significant reduction in morbidity and mortality. As the natural history of the disease is modified, non-communicable diseases are becoming recognized complications of HIV infection, including insulin resistance, type 2 diabetes (DM), and dyslipidemia. A study in the United States revealed that the incidence and prevalence of DM in HIV-infected men with ART exposure was approximately four times greater than that of HIV-seronegative men [1]. While ART suppresses HIV replication, chronic inflammation and immune activation are still ongoing. These are known to increase the risk of metabolic and cardiovascular diseases [2, 3]. Chronic systemic inflammation resulting in dysregulation of glucose and lipid trafficking, utilization, storage [4] and elevated inflammatory biomarker levels is linked to an increased incidence of DM among HIV-infected patients [5].

Prediabetes, a condition in which blood sugars are elevated but not yet meeting the criteria for DM, represents a high risk for future diabetes development. The conversion rate to DM varied among the different populations but was estimated to be between 5 and 11% yearly [6, 7], and up to 70% of people with prediabetes will eventually develop DM [8]. People with prediabetes may have concomitant nephropathies, chronic kidney disease, neuropathies, diabetic retinopathy and macrovascular diseases that are traditionally considered to be complications of diabetes [9–14]. It is well established that diabetes can be prevented or delayed in prediabetes patients using medications such as metformin and/or intensive lifestyle interventions [6, 15, 16]. Caring for patients with prediabetes focused on prevention of the progression to diabetes and minimization of the potential consequences of prediabetes. Therefore, the detection of prediabetes in HIV-infected patients is crucial to identify those who need interventions to reduce future diabetes risk.

The information on prevalence, diagnostic methods, and predicting factors of prediabetes among HIV-infected patients receiving ART are, however, scanty [17]. Previous studies have shown the inaccuracy of hemoglobin A1c (HbA1c) in HIV-infected individuals [18, 19]. The objectives of this study were to determine the prevalence of prediabetes, the agreement of prediabetes diagnostic methods, and its associated factors among HIV-infected individuals receiving ART.

## Methods

### Study design and participants

A cross-sectional study was conducted among HIV-infected adults who received ART and had undetectable plasma viral load (HIV viral load < 40 copies/mL), and followed up at an infectious disease clinic in a university hospital in Bangkok, Thailand. HIV-infected individuals who were receiving ART were consecutively recruited from September 2017 to April 2018. Patients with a history of previously diagnosed DM or using anti-diabetic therapy were excluded. Ethics approvals were obtained from the institutional review boards and informed consent was obtained from participants prior to enrollment. Patient’s information including age, gender, family history of DM in first degree relatives, history of smoking (currently smoking and previous history of smoking), history of alcohol drinking (currently drinking and previous history of drinking), underlying diseases, duration of known HIV infection, ART regimens, duration of ART, CD4 cell counts, anti-hepatitis C virus (HCV) result, serum creatinine and estimated glomerular filtration rate (eGFR) were collected. Hemoglobin (Hb), hematocrit (Hct), and mean corpuscular volume (MCV) were extracted from complete blood count. Anemia was defined as Hb levels < 12.0 g/dL in women and < 13.5 g/dL in men [20]. Hypertension, dyslipidemia, nonalcoholic fatty liver disease, cancer, cerebrovascular disease, coronary artery disease, and chronic kidney disease were categorized as “yes” if the participants had a documented diagnosis of these conditions or were taking medications. The participants were asked if they performed regular physical activity (continuous non-sedentary behavior for more than 10 min that resulted in an increased respiratory rate or heart rate), as well as the duration of sedentary behavior (sitting or reclining, but not include sleeping).

### Anthropometric measurements and laboratory analysis

All anthropometric measurements and blood tests were performed after an overnight fast (8 to 10 h). Each patient was clinically assessed for body weight, body mass index (BMI), neck circumference, waist circumference, and hip circumference. Weight was measured with participants wearing light clothing and no shoes. BMI was calculated as weight (kg)/height^2^ (m^2^). All circumferences were measured to the nearest 0.1 cm using a tape measure. Neck circumference was measured by placing the superior border of the tape measure just below the laryngeal prominence and applied perpendicular to the long axis of the neck. Waist circumference was determined at the umbilical level and hip circumference was measured over non-restrictive underwear at the level of the maximum extension of the buttocks posteriorly on a horizontal plane.

For biochemical parameters, after an overnight fast, a 75 g oral glucose tolerance test (OGTT) was performed. Blood samples were obtained at 0 min for fasting plasma glucose (FPG), HbA1c, lipid profiles, liver function tests, serum creatinine, and at 120 min for 2-hour plasma glucose (2-h PG). The HbA1c assay performed in this study has been certified by the National Glycohemoglobin Standardization Program. Patients were divided into three groups: normal, prediabetes and DM [21]. Prediabetes was defined as FPG between 100 and 125 mg/dL, or 2-h PG between 140 and 199 mg/dL following a 75-g OGTT, or an HbA1c between 5.7% and < 6.5% [21]. DM was defined as an FPG ≥ 126 mg/dL, or a 2-hr PG ≥ 200 mg/dL following a 75-g OGTT, or a HbA1c ≥ 6.5% [21].

### Statistical analysis

A comparison of categorical data was performed using Chi square or Fisher’s exact test. The one-way analysis of variance (ANOVA) or Kruskal–Wallis test was used to compare continuous data between groups. Tukey post hoc test was used to find means that are significantly different from each other. Kappa agreement was computed to estimate the agreement among the three diagnostic definitions. Logistic regression was used to determine the factors associated with prediabetes. Variables that presented a *p*-value < 0.2 from univariate logistic regression were considered in a multivariate logistic regression model. Odds ratio (OR) and its 95% confidence interval (CI) were estimated. All statistical analyses were performed using the Stata statistical software version 15.1.

## Results

### Patient characteristics

A total of 397 HIV-infected patients with a mean age of 47.0 ± 9.8 years were recruited. Of all, 221 (55.7%) patients were male. The mean duration of known HIV infection was 11.9 ± 6.4 years. All patients have received ART with a mean duration of 9.6 ± 5.2 years and had undetectable plasma viral load. The mean CD4 cell count was 554 ± 235 cells/mm^3^. Most patients (77.3%) were receiving non-nucleoside reverse transcriptase inhibitor (NNRTI) as the component of their current ART regimen. Anti-HCV was positive in 11 of 208 patients (5.3%).

Patients were divided into three groups: normal, prediabetes and DM. Of the 397 patients, 133 (33.5%) had prediabetes; 28 (7.1%) had first-diagnosed DM, and the rest had normal glucose tolerance. Mean values of FPG: 2-h PG following a 75-g OGTT in patients with normal, prediabetes, and DM were 89.8 ± 5.7: 98.6 ± 19.0, 99.0 ± 9.9: 130.1 ± 30.3, and 129.5 ± 32.7: 248.2 ± 54.9 mg/dl, respectively. The mean HbA1c in the corresponding groups were 5.2 ± 0.3%, 5.5 ± 0.5%, and 6.4 ± 1.1%, respectively.

Table 1 shows the baseline characteristics of patients in the three groups; normal, prediabetes, and diabetes. There was a significant difference in the mean age among groups. Patients who had normal glucose metabolism were younger than those who had prediabetes (*p* < 0.001) and those who has diabetes (*p *= 0.004). There was no significant difference in age between the prediabetes and diabetes groups (*p *= 0.628). Proportions of patients with hypertension and those with a family history of diabetes were also significantly different among groups (*p* < 0.001, both). The lifestyle of the participants in each group, as collected from self-reported physical activity and hours of sedentary behavior, was not significantly different. The proportions of patients receiving NNRTI and protease inhibitor (PI) were not different among the three groups.

**Table 1:** Baseline characteristics of 397 study patients

Characteristics	Normal (*n *= *236*)	Prediabetes (*n *= *133*)	Diabetes (*n *= *28*)	*P*-value
Age, years, mean ± SD	45.1 ± 10.3	49.4 ± 8.6	51.2 ± 6.9	< .001
Male gender, number (%)	123 (52.1)	82 (61.7)	16 (57.1)	0.206
Family history of diabetes, number (%)	75 (32.3)	42 (32.3)	20 (71.4)	< 0.001
History of smoking, number (%)	75 (31.9)	54 (41.5)	11 (39.3)	0.169
History of alcohol drinking, number (%)	129 (54.9)	89 (68.5)	16 (57.1)	0.039
Regular physical activity, number (%)	193 (82.8)	112 (84.9)	23 (82.1)	0.867
Sedentary behavior, hours per day, median (IQR)	5 (3–8)	5 (2–8)	4 (2–6)	0.340
Underlying diseases, number (%)				
Dyslipidemia	59 (25.1)	47 (35.6)	11 (39.3)	0.054
Hypertension	20 (8.5)	29 (22.0)	12 (42.9)	< 0.001
NAFLD	3 (1.3)	7 (5.3)	1 (3.6)	0.071
Cancer	4 (1.7)	2 (1.5)	2 (7.1)	0.170
Others*	6 (2.5)	5 (3.8)	1 (3.6)	0.559
Duration of HIV infection, years, mean ± SD	11.4 ± 6.6	12.6 ± 5.8	12.9 ± 6.7	0.168
Type of ART regimen, number (%)				
NNRTI-containing	187 (79.6)	94 (70.7)	20 (71.4)	0.284
PI-containing	43 (18.3)	33 (24.8)	8 (28.6)	0.097
Duration of ART, years, mean ± SD	9.1 ± 5.2	10.4 ± 5.1	10.2 ± 5.2	0.079
CD4 cell counts, cells/mm^3^, mean ± SD	549 ± 223	555 ± 247	694 ± 271	0.629
Body weight, kg, mean ± SD	60.6 ± 13.0	62.8 ± 12.2	65.3 ± 10.4	0.076
Body mass index, kg/m^2^, mean ± SD	22.7 ± 3.9	23.4 ± 3.9	24.1 ± 2.8	0.080
Neck circumference, cm, mean ± SD	33.6 ± 3.6	35.1 ± 3.7	36.0 ± 3.7	<0.001
Waist circumference, cm, mean ± SD	81.2 ± 9.9	84.5 ± 11.1	87.6 ± 7.1	<0.001
Hip circumference, cm, mean ± SD	93.2 ± 7.0	94.7 ± 9.5	94.8 ± 5.5	0.178
Alkaline phosphatase, U/L, mean ± SD	94.6 ± 33.8	87.3 ± 32.6	92.6 ± 34.2	0.511
Aspartate transaminase, U/L, mean ± SD	32.4 ± 24.6	34.2 ± 17.1	38.7 ± 16.4	0.681
Alanine transaminase, U/L, mean ± SD	35.1 ± 24.4	42.6 ± 34.8	57.2 ± 41.0	<0.001
Albumin, g/L, mean ± SD	38.9 ± 2.8	38.2 ± 5.5	39.8 ± 2.9	0.485
Total cholesterol, mg/dL, mean ± SD	200 ± 37	202 ± 48	227 ± 62	0.006
HDL-cholesterol, mg/dL, mean ± SD	51 ± 14	48 ± 15	45 ± 9	0.028
LDL-cholesterol, mg/dL, mean ± SD	125 ± 32	123 ± 38	143 ± 52	0.032
Total cholesterol/HDL-cholesterol ratio, mean ± SD	4.1 ± 1.2	4.4 ± 1.5	5.1 ± 1.5	<0.001
Triglycerides, mg/dL, mean ± SD	125 ± 81	186 ± 143	208 ± 122	<0.001
Serum creatinine, mg/dL, mean ± SD	0.8 ± 0.2	0.9 ± 0.3	0.9 ± 0.2	0.017
eGFR, mL/min/1.73 m^2^, mean ± SD	97.6 ± 17.6	91.6 ± 19.2	87.9 ± 17.9	0.001
Hemoglobin, g/dL, mean ± SD	13.5 ± 1.7	13.9 ± 1.8	13.8 ± 2.4	0.104
Hematocrit, %, mean ± SD	39.6 ± 4.6	40.7 ± 5.0	41.0 ± 6.0	0.057
Mean corpuscular volume (fL), mean ± SD	89.4 ± 12.9	90.5 ± 11.7	87.1 ± 12.8	0.407
Anti-HCV positive, number (%) (n = 208)	4 of 128 (3.1)	6 of 64 (9.4)	1 of 16 (6.3)	0.138

ART = antiretroviral therapy; eGFR = estimated glomerular filtration rate; HCV = hepatitis C virus; HDL = high-density lipoprotein; LDL = low-density lipoprotein; NAFLD = nonalcoholic fatty liver disease; NNRTI = non-nucleoside reverse transcriptase inhibitor; PI = protease inhibitor

*Including cerebrovascular disease, coronary artery disease and chronic kidney disease

Pairwise comparisons of the significant results by Tukey post hoc test

Age: normal vs. prediabetes *p *< 0.001; normal vs. diabetes *p *= 0.004; prediabetes vs. diabetes *p *= 0.628

Neck circumference: normal vs. prediabetes *p *< 0.001; normal vs. diabetes *p *= 0.002; prediabetes vs. diabetes *p *= 0.434

Waist circumference: normal vs. prediabetes *p *= 0.011; normal vs. diabetes *p *= 0.006; prediabetes vs. diabetes *p *= 0.316

Alanine transaminase: normal vs. prediabetes *p *= 0.050; normal vs. diabetes *p *= 0.001; prediabetes vs. diabetes *p *= 0.048

Total cholesterol: normal vs. prediabetes *p *= 0.924; normal vs. diabetes *p *= 0.004; prediabetes vs. diabetes *p *= 0.012

HDL-cholesterol: normal vs. prediabetes *p *= 0.133; normal vs. diabetes *p *= 0.070; prediabetes vs. diabetes *p *= 0.492

LDL-cholesterol: normal vs. prediabetes *p *= 0.846; normal vs. diabetes *p *= 0.042; prediabetes vs. diabetes *p *= 0.025

Total cholesterol:/HDL-cholesterol: normal vs. prediabetes *p *= 0.052; normal vs. diabetes *p* < 0.001; prediabetes vs. diabetes *p *= 0.038

Triglyceride: normal vs. prediabetes *p* < 0.001; normal vs. diabetes *p* < 0.001; prediabetes vs. diabetes *p *= 0.605

Serum creatinine: normal vs. prediabetes *p *= 0.021; normal vs. diabetes *p *= 0.289; prediabetes vs. diabetes *p *= 0.999

eGFR: normal vs. prediabetes *p *= 0.008; normal vs. diabetes *p *= 0.023; prediabetes vs. diabetes *p *= 0.602

For anthropometric measurements, neck and waist circumferences were significantly different among groups. Patients who had normal glucose metabolism had lower neck circumference than those who had prediabetes (*p* < 0.001) and those who had diabetes (*p *= 0.002). There was no significant difference in neck circumference between prediabetes and diabetes groups (*p *= 0.434). Also, patients who had normal glucose metabolism had lower waist circumference than those who had prediabetes (*p *= 0.011) and those who had diabetes (*p *= 0.006). There was no significant difference in neck circumference between prediabetes and diabetes groups (*p *= 0.316).

For laboratory investigations, alanine transaminase, low-density lipoprotein (LDL)-cholesterol, total cholesterol/high-density lipoprotein (HDL)-cholesterol ratio, and triglycerides were significantly different among the three groups. Their levels were the greatest in the DM group and lowest in the normal group. Patients who had normal glucose metabolism had lower triglycerides than those who had prediabetes and those who had diabetes (*p* < 0.001, both). There was no significant difference in triglycerides between prediabetes and diabetes groups (*p *= 0.605). eGFR was significantly different among groups. Patients who had normal glucose metabolism had higher eGFR than those who had prediabetes (*p *= 0.008) and those who had diabetes (*p *= 0.023). There was no significant difference in eGFR between prediabetes and diabetes groups (*p *= 0.602). There was no statistically significant difference among groups regarding Hb, Hct, and MCV (*p* > 0.05, all). Anti-HCV was previously performed in 208 of all patients. There was no statistically significant difference among groups (*p *= 0.138).

### Measure of agreement

**Fig. 1:**
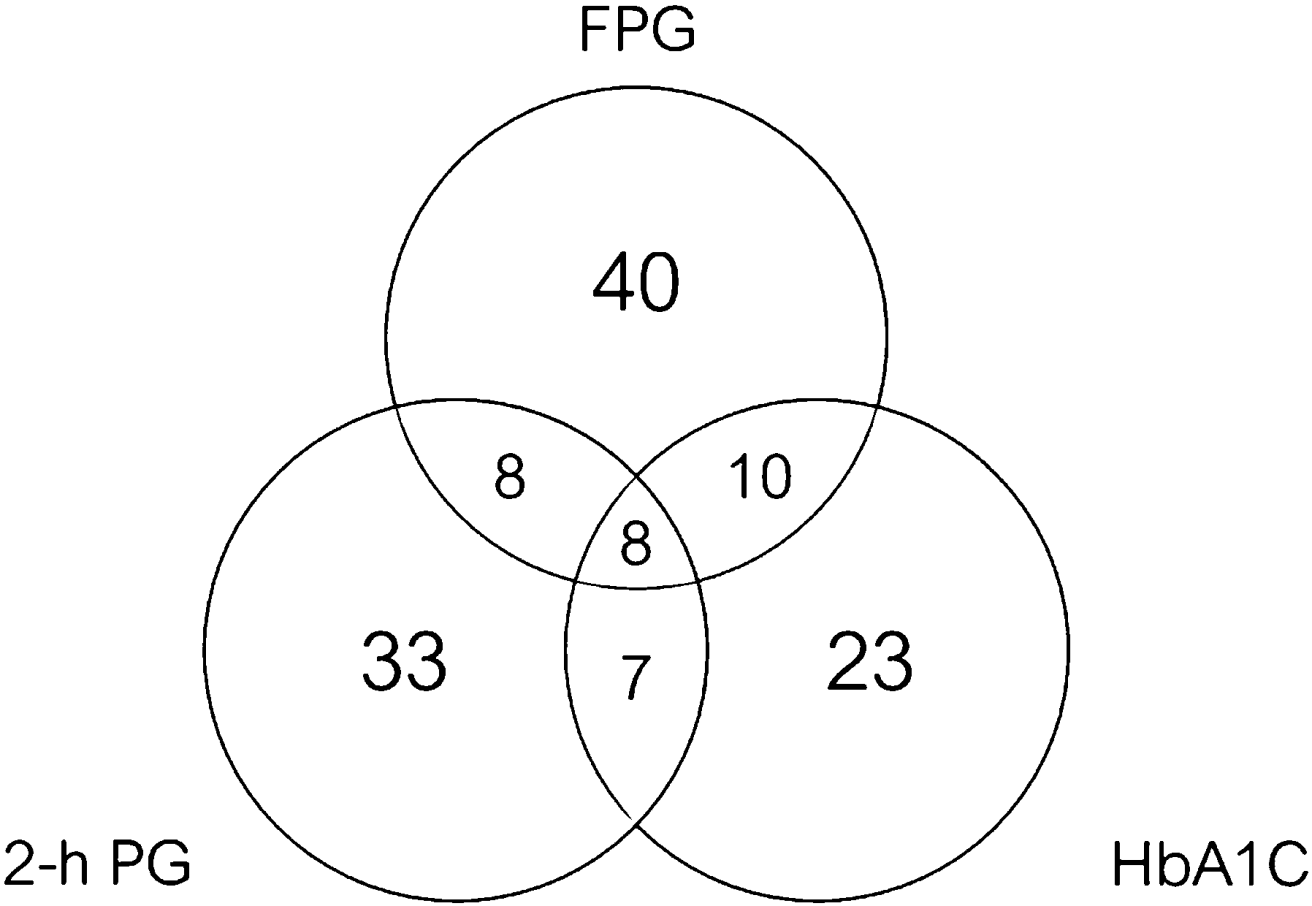
Diagnosis of prediabetes in HIV-infected individuals using fasting plasma glucose (FPG), 2-hour plasma glucose (2-h PG) after performing 75-g oral glucose tolerance test (OGTT), and hemoglobin A1c (HbA1c)

Of those with prediabetes who completed all FPG, 2-h PG, and HbA1C measurements (n = 129), eight cases (6.2%) were determined by all three methods (Fig. 1). Eight cases (6.2%) were determined by FPG and 2-h PG, 7 cases (5.4%) were determined by 2-h PG and HbA1c, and 10 cases (7.8%) were determined by FPG and HbA1c. The diagnosis of prediabetes was determined by FPG alone in 40 cases (31.0%), 2-h PG alone in 33 cases (25.6%), and HbA1c alone in 23 cases (17.8%).

Tables 2, 3 and 4 show analysis of the agreement between FPG and 2-h PG, HbA1c and 2-h PG, and HbA1c and FPG, respectively. Kappa coefficients showed *p *< 0.001 in all pairs of the analysis. Kappa coefficient values were 0.317, 0.429, and 0.396 for the estimate of agreement between FPG and 2-h PG, HbA1c and 2-h PG, and HbA1c and FPG, respectively. After the exclusion of persons with anemia, MCV < 80 or > 100 fL, or eGFR < 60 mL/min/1.73 m^2^, kappa coefficients values were 0.438 and 0.299 for the estimate of agreement between HbA1c and 2-h PG, and HbA1c and FPG, respectively.

**Table 2:** Analysis of the agreement between fasting plasma glucose and 2-hour plasma glucose (2-h PG) after performing 75-g  oral glucose tolerance test (OGTT)

	2-h PG			Total
	< 140	140–199	≥ 200	
FPG				
< 100	249	40	4	293
100–125	51	16	0	67
≥ 126	0	0	21	21
Total	300	56	25	381

*2-h PG* 2-hour plasma glucose, *CI* confidence interval, *FPG* fasting plasma glucose

Kappa coefficient = 0.317; 95% CI 0.241–0.376 (*P* < 0.001)

**Table 3:** Analysis of the agreement between HbA1C and 2-hour plasma glucose (2-h PG) after performing 75-g oral glucose tolerance test (OGTT)

	2-h PG			Total
	< 140	140–199	≥ 200	
HbA1c				
< 5.7	263	41	0	304
5.7–6.4	33	15	0	48
≥ 6.5	0	0	25	25
Total	296	56	25	377

*2-h PG* 2-hour plasma glucose, CI confidence interval, *HbA1c* hemoglobin A1c

Kappa coefficient = 0.429; 95% CI 0.360–0.519 (*P* < 0.001)

**Table 4:** Analysis of the agreement between hemoglobin A1c and fasting plasma glucose

	FPG			Total
	< 100	100–125	≥ 126	
HbA1c				
< 5.7	266	50	0	316
5.7–6.4	31	18	0	49
≥ 6.5	4	0	24	28
Total	301	68	24	393

*CI* confidence interval, *FPG* fasting plasma glucose, *HbA1c* hemoglobin A1c

Kappa coefficient = 0.396; 95% CI 0.356–0.411 (*P* < 0.001)

### Factors associated with prediabetes

When considering patients with prediabetes, univariate logistic regression was performed to investigate factors associated with prediabetes. On univariate logistic regression, age > 45 years, alcohol drinking, hypertension, dyslipidemia, BMI ≥ 25 kg/m^2^, neck circumference > 35 cm, waist circumference > 85 cm, alanine transaminase > 40 U/L, HDL-cholesterol < 40 mg/dL and triglycerides > 150 mg/dL, eGFR, Hb, and positive anti-HCV were factors associated with prediabetes (Table 5). On multivariate logistic regression of factors associated with prediabetes, hypertension (OR 2.22; 95% CI 1.16–9.91; *p *= 0.026), and triglycerides > 150 mg/dL (OR 1.98; 95% CI 1.01–4.44; p = 0.047) were factors significantly associated with prediabetes (Table 5).

**Table 5:** Univariate and multivariate analysis of factors associated with prediabetes

Factors	Univariate analysis			Multivariate analysis		
	OR	95% CI	*p* value	OR	95% CI	*p* value
Age > 45 years old	2.03	1.29–3.17	0.002	1.89	0.84–4.25	0.127
Alcohol drinking^A^	1.78	1.14–2.80	0.012	1.44	0.68–3.04	0.337
Hypertension	3.03	1.63–5.61	< 0.001	3.38	1.16–9.91	0.026
Dyslipidemia	1.65	1.04–2.62	0.034	1.46	0.69–3.09	0.327
Body mass index ≥ 25 kg/m^2^	1.65	1.02–2.66	0.041	1.06	0.39–2.88	0.916
Neck circumference > 35 cm	2.33	1.48–3.65	< 0.001	1.34	0.52–3.48	0.548
Waist circumference > 85 cm	2.19	1.40–3.44	0.001	0.93	0.33–2.60	0.887
ALT > 40 U/L	1.79	1.13–2.84	0.013	1.54	0.71–3.35	0.273
HDL cholesterol < 40 mg/dL	1.64	1.00–2.70	0.050	1.11	0.45–2.75	0.814
Triglycerides > 150 mg/dL	2.72	1.74–4.26	< 0.001	2.11	1.01–4.44	0.047
eGFR	0.98	0.97–0.99	0.003	0.99	0.96–1.01	0.183
Hemoglobin	1.14	1.01–1.29	0.037	0.98	0.79–1.22	0.856
Positive anti-HCV	3.21	0.87–11.80	0.080	3.98	0.98–16.24	0.054

^a^Currently drinking or the previous history of drinking

*ALT* alanine transaminase, *CI* confidence interval, *eGDR* estimated glomerular filtration rate, *HCV* hepatitis C virus, *HDL* high-density lipoprotein, *OR* odds ratio

## Discussion

The current study examined the prevalence, agreement among diagnostic methods, and associated factors of prediabetes among HIV-infected individuals receiving ART in Thailand. The results revealed a strikingly high prevalence of prediabetes at 33.5%. In addition, 7.1% of the study patients were found to have newly diagnosed DM, although FPG had been regularly monitored every 6 months in HIV-infected individuals at our clinic according to the national guidelines. The prevalence of prediabetes in this study appeared to be higher than the 10.6% rate in the general Thai population as revealed by the survey during the same period [22]. Our prevalence rates of prediabetes and DM are comparable to the results from a recent study among HIV-infected individuals in Cameroon (34% with prediabetes and 3.8% with DM) [23], and higher than those in many sub-Saharan African countries [24]. The rates are also higher than our previous study in Thailand in 2006 (impaired fasting glucose and impaired glucose tolerance test of 9.3% and 18.6%, respectively) [17], and in 2009 (27.5% determined by FPG and/or HbA1c) [25], suggesting that the rate of dysglycemia among HIV-infected individuals may be rising.

For the prediabetes diagnostic methods, we observed the interpretation varied throughout the three methods. Our results showed that FPG revealed the highest numbers, whereas HbA1C revealed the lowest numbers of prediabetes diagnosis. This was consistent with previous findings demonstrating lower sensitivity of HbA1C compared to FPG in detecting prediabetes [26, 27]. Overall, the kappa coefficient values of each pairing were 0.3–0.5, which corresponded to minimal to weak agreement of each method [28]. This is similar to a large study in the general Thai population which revealed the kappa statistic for the agreement of FPG and 2 h-PG was 0.55 [29]. In HIV-infected patients, Coelho and coworkers demonstrated poor agreement of prediabetes and DM diagnostic methods [30] with HbA1c being the least sensitive method and OGTT being the most sensitive method. A previous study in non-diabetic persons showed different types of anemia influenced HbA1c differently [31]. Chronic kidney disease (CKD) was also shown to affect HbA1c levels [32, 33]. The kappa coefficient values remained approximately the same after excluding persons with anemia, macrocytosis, microcytosis, and CKD stage 3 or greater. Using FPG alone, prediabetes was missed in 48.8% of our patients, while using FPG and HbA1c increased the detection rate to 74.4%. Performing OGTT routinely might not be practical in clinical settings. However, a recent study demonstrated that random plasma glucose levels below a typical threshold of diabetes were predictive of diabetes development [34]. Such levels have not been explored in prediabetes but could potentially increase the detection rate.

From the multivariate analysis, hypertension and triglycerides > 150 mg/dL were factors significantly associated with prediabetes among HIV-infected individuals. These factors are recognized as risk factors for DM [21]. A recent study from China has shown that hypertension and prediabetes together increase the risk of cardiovascular disease [35]. Triglycerides > 150 mg/dl, common in patients with uncontrolled DM, was another factor independently associated with prediabetes in the present study. A study in Bangladesh had previously demonstrated that high triglycerides were associated with prediabetes as well [36]. HCV infection was known to associate with insulin resistance and prediabetes [37]. In this study, positive anti-HCV showed borderline statistical significance. This might be due to the incomplete data on HCV coinfection.

The strength of our study was the detailed analysis of glycemia estimation using different methods. There were some limitations in the present study. As this was the cross-sectional study, we did not repeat the tests in the absence of unequivocal hyperglycemia according to the criteria for the diagnosis of diabetes recommended by the American Diabetes Association [21]. However, the guidelines suggest that for prediabetes testing, FPG, 2 h-PG, and A1C are equally appropriate. We recruited only HIV-infected patients who received ART with an undetectable plasma viral load. Thus, the results from the present study might not be applicable to the ART-naïve HIV-infected population or HIV-infected patients without successful ART. Since the prevalence of prediabetes varies between different ethnic groups, the results may not be implied for non-Asian populations.

## Conclusions

In summary, we observed the high prevalence of prediabetes among HIV-infected Thai patients receiving ART. The diagnosis methods of prediabetes have minimal to weak agreement. Using FPG together with HbA1c increased the detection rate to approximately 75% of prediabetes patients. Hypertension and triglycerides level over 150 mg/dL are factors significantly associated with prediabetes. HIV-infected patients that present with any of these factors should be assessed for prediabetes and progression to DM.

## Data Availability

The datasets used and/or analyzed during the current study are available from the corresponding author on reasonable request.

## Abbreviations

2-h PG: 2-hour plasma glucose
ANOVA: Analysis of variance
ART: Antiretroviral therapy
BMI: Body mass index
CI: Confidence interval
CKD: Chronic kidney disease
DM: Diabetes
eGFR: Estimated glomerular filtration rate
FPG: Fasting plasma glucose
Hb: Hemoglobin
HbA1c: Hemoglobin A1c
Hct: Hematocrit
HCV: Hepatitis C virus
HDL: High-density lipoprotein
HIV: Human immunodeficiency virus
LDL: Low-density lipoprotein
MCV: Mean corpuscular volume
NNRTI: Non-nucleoside reverse transcriptase inhibitor
OGTT: Oral glucose tolerance test
OR: Odds ratio
PI: Protease inhibitor
